# The roles of social status information in irony comprehension: An eye-tracking study

**DOI:** 10.3389/fpsyg.2022.959397

**Published:** 2022-09-06

**Authors:** Zixuan Wu, Yuxia Wang

**Affiliations:** School of Foreign Languages, Shanghai Jiao Tong University, Shanghai, China

**Keywords:** irony, literality, social status, social skill, eye movement

## Abstract

The literature on irony processing mainly focused on contextual effect, leaving other factors (such as social factors) untouched. The current study investigated how social status information affected the online comprehension of irony. As irony might be more damaging when a speaker uses it to a superordinate than the other way around, it is assumed that greater processing efforts would be observed in the former case. Using an eye-movement sentence reading paradigm, we recruited 36 native Mandarin speakers and examined the role of social status information and literality (i.e., literal and irony) in their irony interpretation. Our results showed ironic statements were more effortful to process than literal ones, reporting an early and consistent effect on the target regions. The social status effect followed the literality effect, with more difficulty in processing ironic statements that targeted the superordinate than the subordinate; such an effect of social status was missing with literal statements. Besides, an individual’s social skill appeared to affect the perception of status information in ironic statements, as the socially skillful readers needed more time than the socially unskillful to process irony targeting a subordinate in the second half of the experiment in the critical region. Our study suggests that irony processing might be further discussed in terms of the relative predictability of linguistic, social, and individual variabilities.

## Introduction

Irony is traditionally viewed as a figurative expression that carries the meaning opposite to its literal meaning, thus violating the Quality Maxim (Grice, 1975). Given the various subtypes (e.g., sarcasm, jocularity, rhetorical questions, hyperbole, and understatement) (Gibbs, 2000), the most common use of irony is to criticize, i.e., ironic criticism infers the negative by stating the positive. It stems from our positive expectations in most cases, so the failure of the expectation may lead to an ironic remark (Wilson and Sperber, 2012). For example, after hiking on a rainy day, a man says “what a good weather!” He is hoping for good weather when he goes hiking. But when the weather is in fact bad, he is complaining about the bad weather by saying the opposite. On account of its typicality, many studies use irony as a synonym for ironic criticism to investigate how irony is produced or comprehended.

The major challenge to understand irony is how to access the speaker’s real intention hidden behind the literal meaning. This has been substantially investigated with three major models discussing how the incongruency between context and the literal meaning leads to the ironic interpretation. The Standard Pragmatic View (Grice, 1975; Searle, 1979), a modular processing model, proposes that the literal meaning is always first activated. As readers notice the incongruency between the context and the literal interpretation, the ironic meaning is then activated, together with the suppression of the former. Following this view, irony comprehension is a more cognitively demanding process, with an extra processing effort following the activation of literal meaning. The Direct Access View (Gibbs, 1986, 2002) states that the context plays a predictive role in the interpretation of the forthcoming expressions, so that when the context is irony-biased, the ironic meaning can be directly activated without necessary full access to the literal meaning. Therefore, no additional effort is required in irony comprehension compared with a literal one. The Gradient Salience Hypothesis (Giora, 1997, 2003; Giora and Fein, 1999) assumes that irony comprehension depends on the meaning of salience (e.g., frequency, conventionality, or familiarity), with the salient meaning being activated prior to other interpretations. Therefore, for conventional ironies, the literal and ironic meanings are equally salient, so that the two meanings can be activated parallelly. However, if the ironic meaning is unconventional, the salient literal meaning is first activated, followed by the access to the ironic interpretation.

The previous experimental studies have yielded mixed results concerning which model better explains the real-time irony processing (e.g., Standard Pragmatic View: Dews and Winner, 1999; Direct Access View: Ivanko and Pexman, 2003; Gradient Salience Hypothesis: Filik et al., 2014). However, when factors other than the context-literal incongruency are considered, that goes beyond the scope of the above three models. In view of this, Katz (2005) and Pexman (2008) have proposed the Constraint-Satisfaction Model, in which all available cues or constraints (e.g., familiarity, language experience, and prosody) are involved and compete in a parallel manner, and the human parser finally reaches the interpretation that best satisfies the available constraints. Hence, if the ironic meaning is supported by more constraints in comparison with the literal meaning, the expression will be interpreted ironically.

There is ample evidence that in addition to the incongruency between contextual valence and literal meaning, other cues, social factors in particular, might constrain the interpretation of irony. For example, as irony normally conveys a critical attitude toward the addressee, it should be used with great caution. In some situations, irony distances the speaker from the addressee when the addressee realizes what he/she is expected to do is in contrast with what he/she actually does; it also seems to maintain or promote relationship as it brings humor or emphasizes solidarity in a conversation (Dews et al., 1995; Jorgensen, 1996; Gibbs and Colston, 2001). Therefore, the speaker normally evaluates the relationship with the addressee to make sure that irony can be understood properly, and irony conducted in an inappropriate relationship may be perceived as somewhat offensive or even assaulting.

There are emerging studies to examine the role of social information in irony comprehension. For example, the addressee can be habituated to how ironic a speaker is and adopt the communicative style to comprehend the ironic utterance (Regel et al., 2010). Besides, children’s understanding of irony that violates socially shared norms can improve reliably better than understanding that violates situationally defined norms (Massaro et al., 2014) with the increase in their age. It is hypothesized that the socially relevant information may be processed with the involvement of the right anterior superior temporal gyrus (Akimoto et al., 2014). As for the relationship between the communicators in an ironic scenario, previous studies mainly focused on the common ground shared by the communicators, namely, “the solidarity relation.” They found the use of irony was deemed more appropriate when the speaker and the addressee share more common ground, such as friends, siblings, or couples (Jorgensen, 1996; Kreuz et al., 1999; Pexman and Zvaigzne, 2004). The literature has generally suggested that a higher degree of solidarity relation might bring a facilitation effect on irony comprehension (Kreuz and Link, 2002), especially at the early processing stage (Whalen et al., 2020). Yet, such a facilitation effect was missing in Pexman et al. (2010). Given the controversial results, further studies are needed to address the effect of social relations, especially when the addresser and the addressee are of unequal social status.

Social status, as a power relation of one speaker over the other (Brown and Gilman, 1960), plays a role in irony production and comprehension: irony is normally directed at a subordinate (Dews et al., 1995) and is deemed inappropriate if used with a superordinate (Jorgensen, 1996). The inappropriateness of irony may damage the relationship between the communicators. Social status plays an important role in Chinese culture, where communicators need to adjust their speech based on their social status in order to maintain politeness since ancient times (Gu, 1990). For example, in Modern Chinese, the second-person singular pronoun *nin* is an honorific pronoun addressing a respected, higher-status addressee, so the inappropriate overuse of *nin* to a subordinate can serve ironic purposes (Chao, 1956; Jiang et al., 2013; Ji, 2021). In a study by Jiang et al. (2013), a more prominent N400 and late positivity effect was reported when a superordinate used *nin* to a subordinate than the other way around, suggesting that Chinese readers had expectations over the use of honorifics, had integration difficulty when the honorifics fail to match the actual social status, and worked hard to figure out the pragmatic intent behind the deliberate misuse of honorifics. Similar effects are also observed with grammatically encoded honorific forms used in some languages, for example, Japanese. The use of Japanese status-inconsistent honorifics can also have an ironic flavor, and an ironic expression with honorific grammar targeting a subordinate is perceived as more ironic and offensive than irony without honorifics (Okamoto, 2002), suggesting that the perception of irony is influenced by status information. Also in Polish, irony initiated by a subordinate to a superordinate is considered more critical and offensive than that conducted in a high-to-low status direction, showing that there might be a culturally independent social norm regarding the risk of using irony to a superordinate (Gucman, 2016). However, the existing studies mainly employed offline methods, e.g., Likert scales, to reveal the effect of social status relations; little is known concerning the online effect of social status information in irony processing.

Apart from social factors, individual differences, social skills in particular, might bear relevance with irony processing. In a self-paced reading study, Spotorno and Noveck (2014) adopted Baron-Cohen et al.’s (2001) Social Skill subscale in Autism Spectrum Quotient (AQ). They divided trials into two halves in terms of the presentation order and found that the socially unskillful participants tended to maintain the reading time difference between literal and ironic sentences, while the socially skillful gradually narrowed the reading time gap in the second half of the experiment (Experiment 2). To examine whether Chinese native readers perform in a similar manner, we also adopt the Social Skill subscale. The subscale contains 10 self-evaluation items, with higher scores indicating lower social skills. It also has the highest internal consistency reliability among all the five subscales (i.e., Social Skill, Attention Switching, Attention to Detail, Communication, and Imagination) in AQ (Austin, 2005; Hurst et al., 2007). Meanwhile, this subscale can also be an indirect measurement of the Theory of Mind, a mechanism underlying the social skill that might infer mental states (Premack and Woodruff, 1978; Baron-Cohen et al., 1985), which are widely examined in irony studies (Dews and Winner, 1997; Wang et al., 2006; Li et al., 2013).

The present study investigates how social status information affects online irony comprehension. As previous studies have shown the inappropriateness of irony used to address a superordinate relative to a subordinate (Jorgensen, 1996; Okamoto, 2002), the study examines the role played by the status information in irony comprehension, especially when the time window unfolds. By adopting the eye-tracking reading paradigm, it is predicted that a longer reading time is needed to process irony targeting a higher-status addressee than a lower-status one. Meanwhile, the literal statements in the baseline condition mainly serve complementary purposes. Based on a survey showing that in Chinese culture, compliments are mostly conveyed toward a status-equal addressee (84.4%), with relatively fewer cases conveyed among the status-unequal (to a subordinate: 10.7%; to a superordinate: 4.9%) (Yu, 2005). It is assumed that the reading time for literal statements might not be significantly affected by unequal status relations.

More importantly, the present study can help distinguish different theoretical accounts of irony comprehension: comparable reading time in processing irony and literal statements supports the Direct Access account (Gibbs, 1986), while longer reading time in the later stage of processing irony statements agrees with the Standard Pragmatic View (Grice, 1975; Searle, 1979). The Constraint-Satisfaction Model (Katz, 2005; Pexman, 2008) can be endorsed if both literality and status information are involved earlier in processing. Meanwhile, the present study follows Spotorno and Noveck (2014) to use the Social Skill subscale, with the purpose to understand how readers’ social skill affects real-time irony processing.

## Materials and methods

### Participants

Thirty-six subjects participated in the study. They were students at Shanghai Jiao Tong University (14 men, aged 19–27 years, mean age = 22.79 years, SD = 2.91; 22 women, aged 18–30 years, mean age = 22.55 years, SD = 2.85). All the participants were native Mandarin speakers born and raised in mainland China, using simplified Chinese as their daily reading and written language. They were all right-handed and had normal or corrected-to-normal vision. None of them reported language or hearing disorders. Participants were recruited in a voluntary manner *via* an online notice and signed a written consent prior to their participation.

### Materials and design

Thirty-two sets of target items were designed for the present study. Each item followed a six-clause structure (see Table 1 for an example). The first clause introduced the background or topic of the scenario. As irony can be normally invited by expectation failure (Kumon-Nakamura et al., 1995; Campbell and Katz, 2012), the second and third clauses used numeric scales to show how the expectation was satisfied or violated, so that the context was literality-biased or irony-biased. The fourth clause revealed the social status relationship between the communicators, so as to manipulate the social relationship between the speaker and the addressee (high-to-low vs. low-to-high). The strategy marking the social status of the communicators was adopted from a study by Jiang et al. (2013). The fifth clause was a literally positive statement made by the speaker, having the linguistic structure of second-person pronoun *ni* + verb + adverb *de* + degree modifiers *zhenshi tai* + evaluative adverb + sentence-final particle *le*. This clause can be interpreted as literal when the context was positive (literality-biased), or ironic when the preceding context was negative (irony-biased). The sixth clause was an attitude-neutral clause, in which the first five characters served as the spill-over region for analysis. Hence, the study had a 2 (literality: literal vs. ironic) × 2 (status: high-to-low vs. low-to-high) within-subject design.

**TABLE 1:** Examples of test items in eye-tracking experiment.

Type	Item
**Literal**	
High-to-low	刘先生陪汪老板射箭，大家大多射了五六环，刘先生射了九十环，汪老板对刘先生说：“你射得/真是太准了！”_critical_/并想想自己 _spill–over_/要怎样射得准一些。 Mr. Liu is shooting arrows with his boss Wang. People normally shoot for five or six points, while Mr. Liu normally shoots for nine or ten points. Boss Wang says to Mr. Liu: “You shoot/so precisely!”_critical_/and starts to think of _spill–over_/how he can shoot more precisely.
Low-to-high	刘先生陪汪老板射箭，大家大多射了五六环，汪老板射了九十环，刘先生对汪老板说：“你射得/真是太准了！” _critical_/并想想自己 _spill–over_/要怎样射得准一些。 Mr. Liu is shooting arrows with his boss Wang. People normally shoot for five or six points, while Boss Wang normally shoots for nine or ten points. Mr. Liu says to Boss Wang: “You shoot/so precisely!”_critical_/and starts to think of _spill–over_/how he can shoot more precisely.
**Ironic**	
High-to-low	刘先生陪汪老板射箭，大家大多射了五六环，刘先生射了一两环，汪老板对刘先生说：“你射得/真是太准了！” _critical_/并想想自己 _spill–over_/要怎样射得准一些。 Mr. Liu is shooting arrows with his boss Wang. People normally shoot for five or six points, while Mr. Liu normally shoots for one or two points. Boss Wang says to Mr. Liu: “You shoot/so precisely!”_critical_/and starts to think of _spill–over_/how he can shoot more precisely.
Low-to-high	刘先生陪汪老板射箭，大家大多射了五六环，汪老板射了一两环，刘先生对汪老板说：“你射得/真是太准了！” _critical_/并想想自己 _spill–over_/要怎样射得准一些。 Mr. Liu is shooting arrows with his boss Wang. People normally shoot for five or six points, while Boss Wang normally shoots for one or two points. Mr. Liu says to Boss Wang: “You shoot/so precisely!”_critical_/, and starts to think of_spill–over_/how he can shoot more precisely.
Question	他们玩的是标枪吗？ Are they throwing javelins?
**Filler**	宋先生去北京玩，朋友请他吃烤鸭，烤鸭一上桌，宋先生对朋友说：“这烤鸭真是太香了！”便立刻夹了一筷子。 Mr. Song is visiting Beijing. His friends invite him to have Beijing roast duck. When the duck is served, Mr. Song says to his friends: “The duck smells so good!” Then he has a taste.
Question	朋友请宋先生吃的是驴打滚吗？ Does the friend treat Mr. Song to rolling donkey*?

*Rolling donkey: a snack in Beijing, consisting of glutinous rice rolls covered by bean flour.

Two validation tests were conducted. A status validation test was conducted to examine the readers’ perception of social status relationships: 12 participants who did not participate in the eye-tracking experiment were instructed to identify the one with higher social status among communicators in each item. Items were counterbalanced across conditions and presented in four lists, with each participant reading one condition within each item. A score of 1 would be given for each item if a participant chose the presumed communicator as having higher status, so the highest score for each item would be 12. Results showed that the average score of status identification was 9.97 (range: 5–12, SD = 1.71), higher than chance level (*t* = 13.106, *p* < 0.001). Besides, an additional 12 participants who did not participate in the eye-tracking experiment rated on a 5-point Likert scale the topic familiarity, smoothness, and scenario rationality of the test items, with 1 coded as “very unfamiliar/unsmooth/irrational” and 5 coded as “very familiar/smooth/rational.” Items were also counterbalanced and presented in four lists, so that each participant only read one condition within each item. The overall familiarity, smoothness, and rationality were 3.96 (SD = 1.12), 3.41 (SD = 1.23), and 2.99 (SD = 1.49), respectively.

Test items were counterbalanced and divided into four lists, so that each list included an equal number of items of the four conditions, and participants would only read one condition within each item. Apart from 32 test items, 70 filler items with a similar six-clause structure were designed and added to each list. They included five types of scenarios: (1) evaluative (*N* = 20): similar to test items, the statement made by the speaker was evaluative, but there was no positive or negative context with numeric comparisons; (2) episodic (*N* = 20): daily communication episodes or Q&As; (3) scalar (*N* = 10): the scalar, numeric comparisons remained in the context, but no evaluative judgment was involved in the commentary clause; (4) comfort (*N* = 10): the context was negative through scalar comparisons but the statement made by the speaker was a comforting expression, and (5) dissatisfaction (*N* = 10): the context was positive through scalar comparisons but the statement made by the speaker showed his/her dissatisfaction toward the addressee. These fillers were added to minimize possible prediction of the literality of statements as participants got familiarized with the experimental procedure. All participants read the same filler items, so there were 32 test items plus 70 filler items for each list.

### Procedure

Participants were tested individually in a sound-proof room. Eye movements were recorded through SR Eyelink 1000, with a sampling rate of 1,000 per second. Only the right eye was recorded. Materials were presented on a 21.5-in monitor (dpi: 1,024 × 768, refresh rate: 100 Hz) 73 cm from the eyes. Prior to the eye-tracking experiment, participants were instructed to read the text on the monitor at their normal reading rate, and complete comprehension questions upon finishing reading. Participants were seated in front of the monitor with their heads positioned on the chin and forehead rest to minimize head movements. After the 9-point calibration procedure, a fixation point would occur on the left quadrant at the start of each trial. Text materials were presented when participants fixated on the point. If their fixation did not match with the point, they were required to have recalibration. Once they completed reading each trial, they pressed the space bar, and a yes-or-no comprehension question appeared on the screen. Participants were asked to answer the question based on the content of the text. Half of the correct answers in each list were “yes” and half were “no.” Feedback was given in each trial to maintain the attention of the participants, and to help the experimenter remind the participants if they provided incorrect answers in consecutive trials. Data were considered valid for a participant when his/her overall accuracy of the response to the comprehension questions was above 75% (1.5 times above chance, Geng et al., 2020). To familiarize the experiment procedure, participants first conducted a practice session consisting of three practice items, which were similar to filler items. In the formal experiment, the whole items were presented in a pseudo-random manner to avoid the consecutive presentation of test items, and the first two trials presented were always filler items. Each character was displayed in a 26-point Song typeface and subtended at about 1° visual angle. Triple spacing was adopted in the presentation.

After the eye-tracking experiment, participants were required to complete the Social Skill subscale (Baron-Cohen et al., 2001) online to assess their social skill performance. The subscale was excerpted from Baron-Cohen et al.’s AQ, an assessment consisting of five subscales: Social Skill, Attention Switching, Attention to Detail, Communication, and Imagination. The Chinese translation was provided with English originals attached for reference.^1^ Each participant only completed the Social Skill subscale of AQ to investigate the relationship between irony understanding and participants’ social skills.

### Data analysis

Two target regions were involved in the analysis, as shown in Table 1. The critical region was formed by a part of the commentary statements that disambiguated literal or ironic interpretations. The spill-over region was the five characters following the critical region, as the reading time difference in the critical region may influence the processing of subsequent words (Shvartsman et al., 2014). For each region, four reading time measures (in milliseconds) were included: first fixation duration (the duration of the first fixation within the current region), gaze duration (or first-pass fixation duration, the sum of the fixation duration of the first run within the current region before the fixation point moves out of the region), regression path duration (the sum of fixations within the current region and the fixations in the prior regions if re-reading occurs in the current region), and total reading time (the sum of all fixation durations within the current region during the entire reading process). These measures showed the possible time course of processing differences between literal and ironic expressions. Specifically, first fixation duration and gaze duration revealed the early processing of the text. Regression path duration showed the difficulty to integrate the words with the current interpretation, and total reading time provided the general processing difficulty of the region. In the preprocessing stage, fixations under 80 ms or above 1,200 ms were filtered, and fixations from 80 to 140 ms were merged with the neighboring fixations. Trials were eliminated if the first fixation duration in the current region of analysis was zero. This procedure removed 9.29% of the data in the critical region, as well as 12.59% in the spill-over region. Logarithmic transformation of the reading time durations was applied in the further analysis to obtain generally normally distributed residuals. Fixations were further trimmed if the standard residual fixation time in the current region was over 2.5. For the critical region, this trimming procedure consisted of 2.20% in first fixation duration, 1.82% in gaze duration, 2.68% in regression path duration, and 1.72% in total reading time of the remaining data. For the spill-over region, it covered 2.78% in first fixation duration, 2.38% in gaze duration, 2.18% in regression path duration, and 1.79% in total reading time.

Analyses of literality and status effects were conducted for the four measures in both critical and spill-over regions, using linear mixed effects (LME) models *via* the *lme4* package (Bates et al., 2014) in R (Version 1.3.1093), with literality (literal vs. ironic), status (high-to-low vs. low-to-high), or their interaction as fixed effects, plus item and participant as random effects. Effect size (partial eta-squared) was calculated using the *effectsize* package (Ben-Shachar et al., 2020). Following Spotorno and Noveck (2014), data were reanalyzed with the interaction between participant’s performance on the Social Skill subscale and literality as a fixed effect, and item as a random effect. This aimed to examine whether individual social skill affects the reading time in ironic relative to literal condition. Besides, if a status effect was reported for irony, analyses of the social skill effect would be conducted to examine whether social skill played a role in different status information within ironic trials. Apart from examining the social skill effect on the overall data, the whole trials were divided into two halves based on the order of presentation (i.e., trials 1–51 and 52–102). This was in line with Spotorno and Noveck (2014) to investigate whether an early-late effect can be reported as the experiment proceeded.

## Results

**FIGURE 1:**
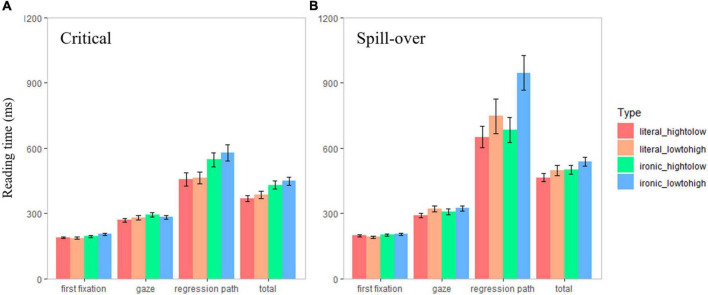
Reading times for the literal and ironic expressions in high-to-low or low-to-high conditions in critical **(A)** and spill-over **(B)** regions. The x-axis represents the reading time measures and conditions, and the y-axis represents the mean value of reading times (in milliseconds). The error bar shows the standard error.

The mean accuracy of correct answers to the comprehension question for each participant was 94.93%, all over the presupposed 75%, suggesting that they all completed the eye-tracking experiment with attention. Figure 1 shows the mean and standard error of reading times for each condition in each target region. The summary of models is presented in Table 2.

**TABLE 2:** Summary of linear mixed models.

		Critical region					Spill-over region				
		B	SE	t	*p*	η^2^_*p*_	B	SE	t	*p*	η^2^_*p*_
First fixation duration (ms)	Literality	0.07	0.02	2.99	0.003	0.009	0.04	0.02	1.77	0.078	0.003
	Status	0.01	0.02	0.56	0.579	0.000	< −0.01	0.02	–0.09	0.929	0.000
	Literality * Status	0.05	0.05	1.20	0.231	0.002	0.08	0.04	1.88	0.061	0.004
	Status in literal	–0.01	0.03	–0.46	0.644	0.000	–0.04	0.03	–1.38	0.169	0.002
	Status in ironic	0.04	0.03	1.23	0.220	0.001	0.04	0.03	1.28	0.202	0.002
Gaze duration (ms)	Literality	0.06	0.03	2.18	0.030	0.005	0.03	0.03	0.93	0.351	0.001
	Status	< −0.01	0.03	–0.12	0.905	0.000	0.06	0.03	1.86	0.064	0.004
	Literality * Status	–0.05	0.06	–0.87	0.386	0.001	0.03	0.06	0.73	0.467	0.001
	Status in literal	0.02	0.04	0.52	0.605	0.000	0.03	0.04	0.80	0.424	0.001
	Status in ironic	–0.03	0.04	–0.71	0.480	0.000	0.08	0.04	1.84	0.067	0.003
Regression path duration (ms)	Literality	0.18	0.04	4.21	<0.001	0.020	0.12	0.05	2.39	0.017	0.006
	Status	0.02	0.04	0.50	0.616	0.000	0.10	0.05	1.96	0.050	0.004
	Literality * Status	<0.01	0.08	0.05	0.963	0.000	0.27	0.10	2.61	0.009	0.007
	Status in literal	0.02	0.06	0.33	0.742	0.000	–0.03	0.07	–0.44	0.660	0.000
	Status in ironic	0.02	0.06	0.39	0.696	0.000	0.24	0.07	3.25	0.001	0.010
Total reading time (ms)	Literality	0.13	0.03	3.97	<0.001	0.020	0.09	0.03	2.86	0.004	0.009
	Status	0.01	0.03	0.42	0.675	0.000	0.07	0.03	2.20	0.028	0.005
	Literality * Status	0.05	0.07	0.82	0.414	0.001	0.04	0.07	0.67	0.504	0.000
	Status in literal	–0.01	0.05	–0.27	0.789	0.000	0.05	0.05	1.09	0.276	0.001
	Status in ironic	0.04	0.05	0.89	0.375	0.001	0.09	0.05	2.04	0.041	0.004

### Literality effect and social status effect

#### Critical region

As shown in Table 2, the literality effect was significant in first fixation duration (*B* = 0.07, *t* = 2.99, *p* = 0.003), gaze duration (*B* = 0.06, *t* = 2.18, *p* = 0.030), regression path duration (*B* = 0.18, *t* = 4.21, *p* < 0.001), and total reading time (*B* = 0.13, *t* = 3.97, *p* < 0.001). Ironic expressions required a longer time to process than literal expressions, as shown in the above measurements (literal vs. ironic: first fixation: 188 vs. 201 ms; gaze: 276 vs. 289 ms; regression path: 461 vs. 563 ms; total: 377 vs. 439 ms). The status effect was insignificant in this region. No literality × status interaction was reported.

#### Spill-over region

In regression path duration, significant effects of literality (*B* = 0.12, *t* = 2.39, *p* = 0.017), status (*B* = 0.10, *t* = 1.96, *p* = 0.050), and their interactions (*B* = 0.27, *t* = 2.61, *p* = 0.009) were reported. *Post hoc* pairwise comparisons showed that for irony, low-to-high condition had longer reading time than high-to-low condition (high-to-low vs. low-to-high: 683 vs. 947 ms, *B* = 0.24, *t* = 3.25, *p* = 0.001), while for literal expressions, the reading time difference was insignificant (high-to-low vs. low-to-high: 651 vs. 747 ms, *B* = −0.03, *t* = −0.44, *p* = 0.660). Significant effects of both literality and status were also reported in total reading time. Irony had longer reading time than literal statements (literal vs. ironic: 482 vs. 520 ms, *B* = 0.09, *t* = 2.86, *p* = 0.004), and statements targeting at the one with higher status had longer reading time than lower status (high-to-low vs. low-to-high: 483 vs. 519 ms, *B* = 0.07, *t* = 2.20, *p* = 0.028). The interaction was insignificant in this region.

### Social skill effect

The coefficient alpha (Cronbach’s alpha) for the Social Skill subscale in the present study was 0.768, indicating that the internal consistency reliability is higher than the “adequate” value of 0.7 (Kline, 2016). The average score of the Social Skill subscale was 3.78 (SD = 2.64), a score lower than autistic individuals (Baron-Cohen et al., 2001), suggesting that participants were less likely to have autistic traits.

**FIGURE 2:**
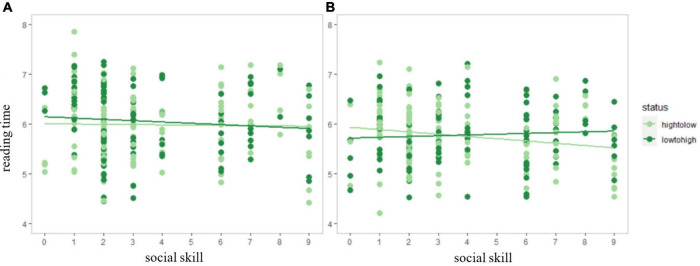
Social skill and logarithmic total reading time of irony in the first **(A)** and the second half **(B)** of the experiment in critical region. The plots represent the data point for each condition (light green: high-to-low, dark green: low-to-high). The light and dark green lines are the regression lines of irony with high-to-low and low-to-high conditions, respectively.

Analyses of linear mixed effect models reported that in both the overall and the halved data, the effect of literality x social skill interaction was insignificant with all the measures in the critical and the spill-over regions (*p*s > 0.05). For status x social skill interaction within irony, the total reading time of the second half of the experiment in the critical region was significant (*B* = 0.06, *t* = 2.218, *p* = 0.027). In this analysis, the reading time data of one participant (social skill scores: 10) were deleted due to a lack of data in irony with the low-to-high conditions. As shown in Figure 2, in the first half of the experiment, reading time did not vary significantly in terms of readers’ social skill performance (high-to-low: *p* = 0.830; low-to-high: *p* = 0.294). *Post hoc* simple linear effect analysis showed that in the second half, readers with lower social skills tended to spend a shorter time than those with higher social skills to process irony targeting a lower status person (*t* = −2.31, *p* = 0.022), while reading time difference remained insignificant for irony with low-to-high condition (*t* = 0.75, *p* = 0.453).

### Bonferroni-corrected significance

As suggested by a reviewer, the multiple comparisons of eye-tracking measures would increase the likelihood of Type I error (Von der Malsburg and Angele, 2017), so corrections of alpha value should be applied. Following the recommendation of von der Malsburg and Angele, Bonferroni correction was conducted to keep the false positivity rate at 0.05. In the present study, four measures in two regions were tested, so that the corrected alpha threshold was 0.00625. Under the strict correction, significant effects of literality were kept for first fixation duration (*p* = 0.003), regression path duration (*p* < 0.001), and total reading time in the critical region (*p* < 0.001), as well as for total reading time in the spill-over region (*p* = 0.004). The effect of status or literality x status interaction was gone for all measures in the two regions. In the analyses of the social skill effect, the status × social skill interaction in total reading time in the second half session turned insignificant when Bonferroni corrections were made.

## Discussion

### Literality effect

By adopting an eye-tracking reading paradigm, the present study aimed to examine the real-time processing of irony, and particularly a time-window analysis of the effects of the literality cue and social status cue. Compared with literal statements, results showed that irony took a reliably longer time to process in the four reading measures in the critical region, indicating that understanding irony is more demanding relative to literal statements. As the reading time for irony in first fixation duration and gaze duration was longer relative to a literal condition in critical regions, readers can immediately perceive the incongruency between the valence of the previous context and the literal meaning of the ironic statements. Besides, the main effect of literality in the regression path duration of both critical and the spill-over regions showed there was an integration difficulty relative to literal statements when the literal meaning did not match the previous context (Filik et al., 2014), and the effect of literality can be consistent.

Results were in line with the Constraint-Satisfaction Model (Katz, 2005; Pexman, 2008), where the contextual constraint came at the early stage of processing. The findings were less compatible with the Direct Access View (Gibbs, 1986, 2002), which predicts comparable processing effort for irony and literal statements. Meanwhile, it was not in accordance with the Standard Pragmatic View (Grice, 1975; Searle, 1979), assuming an extra processing effort of irony after the activation of literal meaning, while the processing difference between irony and literal statements occurred early in the present study. As for the Gradient Salience Hypothesis (Giora, 1997; Giora and Fein, 1999), the present study did not strictly manipulate the salience of ironic meaning. The present study supported an early processing effect of contextual constraint; the question concerning the interaction of the salience of irony and contextual constraint guarantees further studies.

### Social status effect

The interaction of literality and status in the regression path duration in the spill-over region showed the asymmetric effect of social status relation: irony passed in a low-to-high direction required a longer time to process than the other way around, while literal statements had a similar processing time regardless of different social status relationship. Though this did not reach significance when the strict Bonferroni corrections were made, the *p*-value of literality × status interaction was still low (*p* = 0.009), with the status effect kept significant on irony (*p* = 0.001). The result was in line with the prediction in irony comprehension, as irony targeted at a superordinate is less appropriate (Jorgensen, 1996). In this case, readers can perceive the status information and apply the appropriateness of this information in their online reading. Besides, as predicted, the status effect on the comprehension of the literal statement was not found to be significant. This suggested that despite the more frequent occurrence of literal compliments when the recipient is a subordinate (10.7%) than a superordinate (4.9%) (Yu, 2005), readers were insensitive to the status information due to the overall few occurrences of status-unequal compliments. Meanwhile, there might be a general preference for hearing compliments relative to criticism, regardless of social status relation or situational background (Deutsch, 1961). Irony, though milder than literal criticism (Dews et al., 1995; Thompson et al., 2016), still has a damaging effect on account of its critical nature. There was also a significant status effect in the total reading time in the spill-over region, so a statement toward a higher-status person was more difficult to process than toward a lower-status addressee, irrespective of its literality.

Interestingly, the effect of status processing was only observed in the spill-over region, which revealed that the processing of social status information came after the detection of literal/ironic meaning, as the literality effect was already involved in the critical region. The result was similar to a two-stage processing pattern, and the parallel Constraint-Satisfaction Model might be taken into more consideration. In some ERP studies, the comprehension of irony might involve N400- or P600-like (late positivity) effects, where the N400 effect was usually interpreted as the semantic integration between context and literal meaning, while P600 might refer to the pragmatic inference of the ironic intent (Cornejo et al., 2007; Regel et al., 2011; Spotorno et al., 2013; Filik et al., 2014; Caffarra et al., 2019; Mauchand et al., 2021). Despite the fact that the literality and the social status effect occurred in different regions in the current study, the time course of literality and status processing was similar in principle to the N400 and P600 effects found in previous studies. Therefore, it is likely that after readers figured out the ironic nature, they might move on to integrate the status relation to reason the communicative intent or motivation behind the ironic statement.

One possible reason for the later effect of social status was that status information may not be weighted as heavily in the prediction of irony as literality. Literality is mostly an overwhelming factor in ironic interpretation (Deliens et al., 2018), while status information mainly adjusts the degree or appropriateness of irony, having little effect on the literal/ironic judgment. This can be further evidenced in view of the effect significance after Bonferroni correction, where only the literality effect was observed, while the status effect became insignificant for all measures in both regions. Results were not in line with the early effect of sibling relationships reported in children (Whalen et al., 2020). This may be explained that for children, internal state language (e.g., expressions about emotions, beliefs, and desires) constitutes an important part of sibling relations (Howe, 1991), thus making irony, a typical internal state language expressing the belief and intent (Dews and Winner, 1997), possibly more predictable when children receive it from their siblings. Therefore, literality might be privileged in processing relative to status information.

### Social skill effect

As for the individual differences as measured in social skill effect, the present study failed to report any social skill effect in processing ironic vs. literal statements, when analyzed as a whole or into two halves. This was contrary to the findings in Spotorno and Noveck (2014), where social skill played a part in the anticipation of irony as the experiment unfolded: the socially unskillful participants tended to maintain the reading time difference in the second half of the experiment, while the socially skillful performed alike in processing literal and ironic sentences. One possible explanation might be that their study constructed a one-to-one mapping between the negative context and irony, so that the socially skillful can gradually anticipate the occurrence of irony. In the present study, the well-designed filler items (e.g., the comforting statements) obscured the prediction of irony.

As for the interaction between the social skill and the social status for ironic statements, only in the second half of the experiment in total reading time of the critical region, the reading time of irony passed in a high-to-low direction was negatively correlated with AQ scores. That is, those who were socially skillful tended to have longer reading time than the socially unskillful when they read irony directed to a subordinate. This might be attributed to the frequent use of indirect criticism (including irony) in Chinese culture (Tang, 2016; Lin, 2020), and the function of face protection in irony transmitted in a high-to-low direction (Gucman, 2016). Since individuals having higher scores in the Social Skill subscale (i.e., lower social skill competence) are less likely to be extravert and agreeable (Austin, 2005), they might be more welcome or expect such moderate commentary statements when placed in a negative context, thus having shorter reading time than socially skillful readers. Nevertheless, irony toward the superordinate violated the general social norm, so that it was less expected for readers regardless of their social skills. But generally, the social skill effect might be interpreted with caution, given that it was only reported in the second half of the total reading time of the critical region, and these effects turned insignificant when the strict Bonferroni corrections were made. It is possible that the group with richer and more complicated social experiences than the participants in the present study (i.e., university students) may be more sensitive to the status information, thus having a more prominent effect when testing their social skills and irony comprehension. Still, the social skill and status interaction for irony found in the critical region suggested that the time of the involvement of social status processing may vary across participants, as the main effect of status was only reported in the spill-over region. Taken together with the literality and status effects discussed earlier, the relative predictive power of irony caused by available constraints (Deliens et al., 2018; e.g., literality, prosody, facial expression, and sociocultural information) may vary across individuals, and hence the Constraint-Satisfaction Model (Katz, 2005; Pexman, 2008) can be further discussed in terms of the priority of these constraints.

## Conclusion

The current eye-tracking study examined the role that social status information played in the time course of online irony comprehension, also addressing the current processing models on irony. Results showed an early and long-lasting effect of literality, indicating more effortful processing of irony compared with literal statements. The findings are more consistent with the Constraint-Satisfaction Model (Katz, 2005; Pexman, 2008). However, the social status had a delayed effect following the literality effect, with longer reading time for irony targeting a superordinate than at a subordinate, suggesting the violation of social norms would cause processing difficulty, and the predictability of irony from social status cue may not be as powerful as context-literal incongruency (i.e., literality cues). Finally, individual social skills revealed the individual perceptual variation of status information in the critical region in the second half of the trials, indicating that the current processing models shall be further investigated in terms of individual variations.

## Data availability statement

The original contributions presented in this study are included in the article/supplementary material, further inquiries can be directed to the corresponding author.

## Ethics statement

The studies involving human participants were reviewed and approved by Ethics Committee of School of Foreign Languages, Shanghai Jiao Tong University. The patients/participants provided their written informed consent to participate in this study.

## Author contributions

ZW collected and analyzed the data. Both authors collaborated on the experimental design, interpreted the data, conducted manuscript writing, and approved the submitted version.

## Footnotes

1 The Chinese translation of AQ: https://www.douban.com/note/92776422/.

## References

[B1] Akimoto Y. Sugiura M. Yomogida Y. Miyauchi C. M. Miyazawa S. Kawashima R. (2014). Irony comprehension: Social conceptual knowledge and emotional response. *Hum. Brain Mapp.* 35 1167–1178. 10.1002/hbm.22242 23408440 PMC6869100

[B2] Austin E. J. (2005). Personality correlates of the broader autism phenotype as assessed by the Autism Spectrum Quotient (AQ). *Pers. Individ. Dif.* 38 451–460. 10.1016/j.paid.2004.04.022

[B3] Baron-Cohen S. Leslie A. M. Frith U. (1985). Does the autistic child have a “theory of mind”? *Cognition* 21 37–46. 10.1016/0010-0277(85)90022-8 2934210

[B4] Baron-Cohen S. Wheelwright S. Skinner R. Martin J. Clubley E. (2001). The autism-spectrum quotient (AQ): Evidence from asperger syndrome/high-functioning autism, malesand females, scientists and mathematicians. *J. Autism Dev. Disord.* 31 5–17. 10.1023/A:1005653411471 11439754

[B5] Bates D. Mächler M. Bolker B. Walker S. (2014). Fitting linear mixed-effects models using lme4. Available online at: https://arxiv.org/pdf/1406.5823.pdf (accessed May 24, 2022). 10.18637/jss.v067.i01

[B6] Ben-Shachar M. S. Lüdecke D. Makowski D. (2020). effectsize: Estimation of effect size indices and standardized parameters. *J. Open Source Softw.* 5:2815. 10.21105/joss.02815

[B7] Brown R. Gilman A. (1960). “The pronouns of power and solidarity,” in *Style in Language*, ed. Sebeok T. A. (Cambridge: MIT Press), 253–276.

[B8] Caffarra S. Motamed Haeri A. Michell E. Martin C. D. (2019). When is irony influenced by communicative constraints? ERP evidence supporting interactive models. *Eur. J. Neurosci.* 50 3566–3577. 10.1111/ejn.14503 31282038

[B9] Campbell J. D. Katz A. N. (2012). Are there necessary conditions for inducing a sense of sarcastic irony? *Discourse Proc.* 49 459–480. 10.1080/0163853X.2012.687863

[B10] Chao Y. R. (1956). Chinese terms of address. *Language* 32 217–241. 10.2307/410666

[B11] Cornejo C. Simonetti F. Aldunate N. Ibáñez A. López V. Melloni L. (2007). Electrophysiological evidence of different interpretative strategies in irony comprehension. *J. Psycholinguist. Res.* 36 411–430. 10.1007/s10936-007-9052-0 17364233

[B12] Deliens G. Antoniou K. Clin E. Ostashchenko E. Kissine M. (2018). Context, facial expression and prosody in irony processing. *J. Memory Lang.* 99 35–48. 10.1016/j.jml.2017.10.001

[B13] Deutsch M. (1961). The interpretation of praise and criticism as a function of their social context. *J. Abnormal Soc. Psychol.* 62 391–400. 10.1037/h0042264 13722332

[B14] Dews S. Kaplan J. Winner E. (1995). Why not say it directly? The social functions of irony. *Discourse Proc.* 19 347–367. 10.1080/01638539509544922

[B15] Dews S. Winner E. (1997). Attributing meaning to deliberately false utterances: The case of irony. *Adv. Psychol.* 122 377–414. 10.1016/S0166-4115(97)80142-2

[B16] Dews S. Winner E. (1999). Obligatory processing of literal and nonliteral meanings in verbal irony. *J. Pragmat.* 31 1579–1599. 10.1016/S0378-2166(99)00005-3

[B17] Filik R. Leuthold H. Wallington K. Page J. (2014). Testing theories of irony processing using eye-tracking and ERPs. *J. Exp. Psychol.* 40 811–828. 10.1037/a0035658 24548324

[B18] Geng P. Gu W. Johnson K. Erickson D. (2020). “Acoustic-Prosodic and Articulatory Characteristics of the Mandarin Speech Conveying Dominance or Submissiveness,” in *Proc. 10th International Conference on Speech Prosody*, (Tokyo, Japan), 424–428. 10.21437/SpeechProsody.2020-87

[B19] Gibbs R. W. (1986). On the psycholinguistics of sarcasm. *J. Exp. Psychol.* 115 3–15. 10.1037/0096-3445.115.1.3

[B20] Gibbs R. W. (2000). Irony in talk among friends. *Metaphor Symb.* 1 5–27. 10.1080/10926488.2000.9678862

[B21] Gibbs R. W. (2002). A new look at literal meaning in understanding what is said and implicated. *J. Pragmat.* 34 457–486. 10.1016/S0378-2166(01)00046-7

[B22] Gibbs R. W. Colston H. L. (2001). “The risks and rewards of ironic communication,” in *Say not to Say: New Perspectives on Miscommunication*, eds Anolli L. Ciceri R. Riva G. (Amsterdam: IOS Press), 181–193.

[B23] Giora R. (1997). Understanding figurative and literal language: The graded salience hypothesis. *Cogn. Linguist.* 8 183–206. 10.1515/cogl.1997.8.3.183 31158291

[B24] Giora R. (2003). *On our Mind: Salience, Context, and Figurative Language.* New York, NY: Oxford University Press. 10.1093/acprof:oso/9780195136166.001.0001

[B25] Giora R. Fein O. (1999). Irony: Context and salience. *Metaphor Symb.* 14 241–257. 10.1207/S15327868MS1404_1

[B26] Grice H. P. (1975). “Logic and conversation,” in *Speech Acts, Vol. 3: Syntax and Semantics*, eds Cole P. Morgan J. L. (New York, NY: Academic Press), 41–58. 10.1163/9789004368811_003

[B27] Gu Y. (1990). Politeness phenomena in modern Chinese. *J Pragmat.* 14 237–257. 10.1016/0378-2166(90)90082-O

[B28] Gucman M. (2016). The role of individual differences and situational factors in perception of verbal irony. *Psychol. Lang. Commun.* 20 255–277. 10.1515/plc-2016-0016

[B29] Howe N. (1991). Sibling-directed internal state language, perspective taking, and affective behavior. *Child Dev.* 62 1503–1512. 10.2307/1130822 1786731

[B30] Hurst R. M. Mitchell J. T. Kimbrel N. A. Kwapil T. K. Nelson-Gray R. O. (2007). Examination of the reliability and factor structure of the Autism Spectrum Quotient (AQ) in a non-clinical sample. *Pers. Individ. Dif.* 43 1938–1949. 10.1016/j.paid.2007.06.012

[B31] Ivanko S. L. Pexman P. M. (2003). Context incongruity and irony processing. *Discourse Proc.* 35 241–279. 10.1207/S15326950DP3503_2

[B32] Ji L. (2021). When politeness processing encounters failed syntactic/semantic processing. *Acta Psychol.* 219:103391. 10.1016/j.actpsy.2021.103391 34412023

[B33] Jiang X. Li Y. Zhou X. (2013). Is it over-respectful or disrespectful? Differential patterns of brain activity in perceiving pragmatic violation of social status information during utterance comprehension. *Neuropsychologia* 51 2210–2223. 10.1016/j.neuropsychologia.2013.07.021 23916511

[B34] Jorgensen J. (1996). The functions of sarcastic irony in speech. *J. Pragmat.* 26 613–634. 10.1016/0378-2166(95)00067-4

[B35] Katz A. N. (2005). “Discourse and sociocultural factors in understanding nonliteral language,” in *Figurative Language Comprehension: Social and Cultural Influences*, eds Colston H. L. Katz A. N. (Mahwah: Lawrence Erlbaum Associates, Inc), 183–207.

[B36] Kline R. B. (2016). *Principles and Practice of Structural Equation Modeling.* New York, NY: Guilford Publications.

[B37] Kreuz R. J. Kassler M. A. Coppenrath L. Allen B. M. (1999). Tag questions and common ground effects in the perception of verbal irony. *J. Pragmat.* 31 1685–1700. 10.1016/S0378-2166(99)00010-7

[B38] Kreuz R. J. Link K. E. (2002). Asymmetries in the use of verbal irony. *J. Lang. Soc. Psychol.* 21 127–143. 10.1177/02627X02021002002

[B39] Kumon-Nakamura S. Glucksberg S. Brown M. (1995). How about another piece of pie: The allusional pretense theory of discourse irony. *J. Exp. Psychol.* 124 3–21. 10.1037/0096-3445.124.1.3 7897341

[B40] Li J. P. Law T. Lam G. Y. To C. K. (2013). Role of sentence-final particles and prosody in irony comprehension in Cantonese-speaking children with and without Autism Spectrum Disorders. *Clin. Linguist. Phonet.* 27 18–32. 10.3109/02699206.2012.734893 23237415

[B41] Lin C. Y. (2020). Exploring judges’ compliments and criticisms on American, British, and Taiwanese talent shows. *J. Pragmat.* 160 44–59. 10.1016/j.pragma.2020.02.008

[B42] Massaro D. Valle A. Marchetti A. (2014). Do social norms, false belief understanding, and metacognitive vocabulary influence irony comprehension? A study of five-and seven-year-old children. *Eur. J. Dev. Psychol.* 11 292–304. 10.1080/17405629.2013.821407

[B43] Mauchand M. Caballero J. A. Jiang X. Pell M. D. (2021). Immediate online use of prosody reveals the ironic intentions of a speaker: Neurophysiological evidence. *Cogn. Affective Behav. Neurosci.* 21 74–92. 10.3758/s13415-020-00849-7 33420711

[B44] Okamoto S. (2002). Politeness and the perception of irony: Honorifics in Japanese. *Metaphor Symbol.* 17 119–139. 10.1207/S15327868MS1702_3

[B45] Pexman P. M. (2008). It’s fascinating research: The cognition of verbal irony. *Curr. Direct. Psychol. Sci.* 17 286–290. 10.1111/j.1467-8721.2008.00591.x

[B46] Pexman P. M. Whalen J. M. Green J. J. (2010). Understanding verbal irony: Clues from interpretation of direct and indirect ironic remarks. *Discourse Proc.* 47 237–261. 10.1080/01638530902959901

[B47] Pexman P. M. Zvaigzne M. T. (2004). Does irony go better with friends? *Metaphor Symbol.* 19 143–163. 10.1207/s15327868ms1902_3

[B48] Premack D. Woodruff G. (1978). Does the chimpanzee have a theory of mind? *Behav. Brain Sci.* 4 515–526. 10.1017/S0140525X00076512

[B49] Regel S. Coulson S. Gunter T. C. (2010). The communicative style of a speaker can affect language comprehension? ERP evidence from the comprehension of irony. *Brain Res.* 1311 121–135. 10.1016/j.brainres.2009.10.077 19900421

[B50] Regel S. Gunter T. C. Friederici A. D. (2011). Isn’t it ironic? An electrophysiological exploration of figurative language processing. *J. Cogn. Neurosci.* 23 277–293. 10.1162/jocn.2010.21411 20044894

[B51] Searle J. R. (1979). *Expression and Meaning: Studies in the Theory of Speech Acts.* Cambridge: Cambridge University Press. 10.1017/CBO9780511609213

[B52] Shvartsman M. Lewis R. L. Singh S. (2014). “Computationally rational saccadic control: An explanation of spillover effects based on sampling from noisy perception and memory,” in *Proceedings of the Fifth Workshop on Cognitive Modeling and Computational Linguistics*, (Baltimore, MD: Association for Computational Linguistics). 10.3115/v1/W14-2001

[B53] Spotorno N. Cheylus A. Van Der Henst J. B. Noveck I. A. (2013). What’s behind a P600? Integration operations during irony processing. *PLoS One* 8:e66839. 10.1371/journal.pone.0066839 23826155 PMC3691266

[B54] Spotorno N. Noveck I. A. (2014). When is irony effortful? *J. Exp. Psychol.* 143 1649–1665. 10.1037/a0036630 24773194

[B55] Tang C. (2016). Managing criticisms in US-based and Taiwan-based reality talent contests: A cross-linguistic comparison. *Pragmatics* 26 111–136. 10.1075/prag.26.1.06tan 33486653

[B56] Thompson D. Mackenzie I. G. Leuthold H. Filik R. (2016). Emotional responses to irony and emoticons in written language: Evidence from EDA and facial EMG. *Psychophysiology* 53 1054–1062. 10.1111/psyp.12642 26989844 PMC4999054

[B57] Von der Malsburg T. Angele B. (2017). False positives and other statistical errors in standard analyses of eye movements in reading. *J. Memory Lang.* 94 119–133. 10.1016/j.jml.2016.10.003 28603341 PMC5461930

[B58] Wang A. T. Lee S. S. Sigman M. Dapretto M. (2006). Neural basis of irony comprehension in children with autism: The role of prosody and context. *Brain* 129 932–943. 10.1093/brain/awl032 16481375 PMC3713234

[B59] Whalen J. M. Doyle A. Pexman P. M. (2020). Sarcasm between siblings: Children’s use of relationship information in processing ironic remarks. *J. Pragmat.* 156 149–159. 10.1016/j.pragma.2019.05.005

[B60] Wilson D. Sperber D. (2012). “Explaining Irony,” in *Meaning and Relevance*, eds Sperber D. Wilson D. (Cambridge: Cambridge University Press), 123–145. 10.1017/CBO9781139028370.008

[B61] Yu M. C. (2005). Sociolinguistic competence in the complimenting act of native Chinese and American English speakers: A mirror of cultural value. *Lang. Speech* 48 91–119. 10.1177/00238309050480010501 16161474

